# Editorial: Metabolic miscommunication among organs: The missing links

**DOI:** 10.3389/fendo.2023.1136283

**Published:** 2023-01-13

**Authors:** Maria João Meneses, Maria Paula Macedo

**Affiliations:** ^1^ iNOVA4Health, NOVA Medical School, Faculdade de Ciências Médicas, NMS, FCM, Universidade Nova de Lisboa, Lisboa, Portugal; ^2^ DECSIS II Iberia, Évora, Portugal

**Keywords:** diabetes, metabolism, NAFLD, crosstalk, exercise, inflammation

Type 2 diabetes (T2D) is one of the modern metabolic diseases that involve impaired or faulty communication between tissues and/or organs. Extracellular vesicles, endocrine hormones, and cytokines are just some of the communication/signaling molecules that may be affected, involving both commensal microbiota and constitutive tissues. For these processes to be identified and characterized in a clinical setting, various disciplines and methods of analysis and diagnosis must be integrated. As an example, the imaging of liver fat and fibrosis could be analyzed along with the characterization of intestinal microbiome activity to understand the role of intestinal dysbiosis in fatty liver disease development.

The goal of this Research Topic was to present new progress in the integration of epidemiology and pathogenic mechanisms that improve the understanding of biological crosstalk within diabetes and related disorders. Indeed, the authors replied to our call and the manuscripts within this Research Topic include the link between diabetes and intestinal disorders, kidney disease, bone metabolism, nocturia, iron and nitrogen metabolism, exercise as well as inflammation and liver disease.

Meneses et al. studied the impact of high-fat and high-fructose feeding on the metabolome of the liver, muscle, white and brown adipose tissue. By feeding male mice with these diets for 12 weeks, it was possible to disclose that although both diets have deleterious effects on the liver, causing non-alcoholic fatty liver disease (NAFLD), high-fat resulted in a higher impact on hepatic glucose and lipid metabolism than high-fructose, leading namely to glucose intolerance. Moreover, while high-fat had alterations in brown adipose tissue metabolites that indicate increased thermogenesis, high-fructose led to increased levels of betaine, known to be a shielding metabolite against fructose-induced inflammation. Overall, this study indicates that high-fat and high-fructose feeding have a negative but distinct effect on the metabolome of the abovementioned organs.

Besides high circulating lipid and fructose levels, iron levels also impact metabolic homeostasis. Wang et al. aimed at exploring the association of serum iron levels with metabolic dysfunction-associated fatty liver disease (MAFLD) in Chinese patients with T2D in a cross-sectional study with 1,467 individuals. Indeed, the association between iron metabolism and insulin resistance has been explored in the last few years, and the present study found a positive correlation between the presence of MAFLD and serum iron levels in patients with T2D. As such, serum iron levels may act as one indicator for MAFLD risk assessment in those individuals.

The clinical evaluation of circulating and phenotypic characteristics changes with disease progression and aging as observed by Zheng et al. who evaluated the association of liver enzymes with diabetes mellitus risk in different obesity subgroups based on a middle-aged Chinese population. Although serum GGT levels were correlated with diabetes mellitus risk in the middle-aged population, the correlation disappeared when waist circumference was over 98.99 cm.

How organs crosstalk impacts dysmetabolism is a step forward in understanding dysmetabolism. Yan et al. shed the light on the liver-kidney axis by exploring, through machine learning techniques, crosstalk genes in NAFLD and diabetic nephropathy. The authors pinpoint that there seems to be a common pathogenesis between these metabolic disorders and that lipoprotein lipase (LPL) and secreted phosphoprotein 1 (SPP1) are the most relevant crosstalk genes. The contribution of nephropathy for diabetes and nocturia is covered by Fu et al. through a systematic review and meta-analysis. Nocturia is known to be connected to age, but it may be also influenced by hypertension. As there are many confounding factors (e.g., age, gender), the associations need to be carefully performed. Diabetes had a 1.49-fold higher risk of nocturia. The association was stronger for Asian and male subjects or those at a lower nocturia cut-off.

Delgado et al. describe the importance of gut-liver axis nitrogen metabolism for the onset and development of NAFLD. Indeed, the authors depict how disrupted nitrogen metabolism and metabolic miscommunication between the gut and the liver may lead to NAFLD and shed the light on the re-establishment of altered gut-liver axis nitrogenous balance as a possible therapeutic approach to tackle NAFLD.

Yang et al. aimed at determining the impact of glucose levels at admission and during the first week (early phase) on clinical outcomes in patients with acute pancreatitis and to investigate the relationship between stress hyperglycemia and hypertriglyceridemia. Indeed, stress hyperglycemia, both during admission and during the first week of admission worsens the clinical outcomes of patients with acute pancreatitis. These effects were even more noticeable when hypertriglyceridemia co-existed at admission.

Cheng et al. performed a bibliometric analysis from 2000 to 2021 about the link between bone metabolism and diabetes *mellitus* where osteoporosis and associated fractures are the greatest concern in the bone metabolism field. With this publication, it becomes evident that cross-discipline research fields are attracting increased attention. Specifically, these are valuable insights for clinicians to recognize diabetic osteopenia and provide more attention and support to patients at risk of developing osteopenia.

Wang et al. conducted a cross-sectional study to clarify the association between inflammatory indicators and metabolic diseases, and cardiovascular disease risk. Contrary to the neutrophil-to-lymphocyte ratio and systematic immune-inflammation index, the monocyte-to-high-density lipoprotein ratio and systemic inflammation response index had a significant positive association with metabolic diseases and their components. Furthermore, they also correlated with cardiovascular disease, and the increment of these indicators caused a gradually evaluated risk of 10-year cardiovascular disease risk assessed by the Framingham score.

The prevention and disease control was tackled by Houttu et al. who systematically reviewed the effect of aerobic exercise on NAFLD, specifically in non-alcoholic steatohepatitis (NASH) and liver fibrosis. The 24 analyzed studies illustrate that liver fat is decreased by aerobic exercise (moderate-intensity continuous training or high-intensity interval training) with a concomitant decrease of alanine transaminase and aspartate aminotransferase. However, further studies are needed to elucidate the impact of moderate-intensity continuous training versus high-intensity interval training on hepatic inflammation and fibrosis.

Wang et al. provided a commentary on the previously published manuscript by Riedel et al. (1) calling attention to the need to look to T2D beyond hyperglycemia. Indeed, the authors support that T2D is more likely to be a syndrome leading to hyperglycemia, systematic inflammation, insulin resistance, and intestinal bowel disease, indeed a systematic disease as previously supported by Pina et al. (2, 3). As such, hyperglycemia could be treated as a coexistent symptom rather than the central one. We have also shared that opinion in a recent review where we aim to call attention to insulin and C-peptide as central molecules to help in the phenotypic diagnosis of T2D along with glucose, rather than glucose *per se* (4).

In summary, papers on this Research Topic cover a wide spectrum of metabolic miscommunication among organs and underline once more how important is to have a wider view of these disorders and how organs communicate, both trying to compensate or spreading the “disease” message.

## Author contributions

MJM drafted the manuscript. MPM reviewed, provided input, and approved the content.
